# Monitoring the battleground: exploring antimicrobial resistance and virulence factors in wound bacterial isolates

**DOI:** 10.1099/acmi.0.000613.v6

**Published:** 2023-11-09

**Authors:** Silas Onyango Awuor, Eric O. Omwenga, Richard M. Mariita, Jonathan M. Musila, Stanslaus Musyoki

**Affiliations:** ^1^​ Microbiology Department, Jaramogi Oginga Odinga Teaching and Referral Hospital, Kisumu, Kenya; ^2^​ Department of Medical Microbiology & Parasitology, School of Health Sciences, Kisii University, Kisii, Kenya; ^3^​ Microbial BioSolutions, Troy, New York, 12180, USA; ^4^​ Fayetteville State University, Fayetteville, North Carolina, 28301, USA; ^5^​ Department of Medical Laboratory Sciences, School of Health Sciences, South Eastern Kenya University, Kitui, Kenya

**Keywords:** antimicrobial resistance, human pathogens, susceptibility, chronic wounds, virulence factors, ESKAPE pathogens, wound management

## Abstract

Antibiotic resistance poses a grave global public health threat, exacerbated by widespread and often inappropriate antibiotic usage. Vigilant surveillance of antibiotic utilization and emergence of antimicrobial resistance (AMR) is essential. Of particular concern in the era of AMR is the persistent issue of chronic wound infections. To address this, we conducted a comprehensive evaluation of wound isolates from chronic wounds at Jaramogi Oginga Odinga Teaching and Referral Hospital (JOOTRH) in Kenya, to identify relevant bacteria and assess their drug resistance patterns.Wound samples were collected and processed using standard microbiological methods. Bacterial isolates were identified and assessed for their susceptibility to a panel of antibiotics using the Kirby-Bauer disk diffusion method. A total of 103 bacterial isolates were obtained from the wound samples, with a higher prevalence in male patients (59%). *Staphylococcus aureus* (20.7 %) emerged as the most predominant pathogen, followed by *Klebsiella* spp. (14.8 %), *Pseudomonas aeruginosa* spp. (14.8 %) and *Escherichia coli* (4.4 %) in wound samples. High levels of antibiotic resistance were observed among the isolates, with the highest resistance rates reported for cotrimoxazole (48.1 %), clindamycin (25.9 %) and erythromycin (25.9 %). Furthermore, among the isolates, 75 % produced haemolysin and protease, while 50 % produced lipase and phospholipase, factors that enhance virulence and survival. The findings of this study highlight the alarmingly high prevalence of antibiotic resistance among bacterial pathogens isolated from chronic wounds in Kenya. This poses a major challenge to the effective management of chronic wound infections. There is an urgent need to implement effective antimicrobial stewardship programs and develop new antibiotics to combat the growing threat of antibiotic resistance.

## Data Summary

All the data have been shared in this paper. Supplementary material S1, available in the online version of this article, shows isolate growth on blood agar media: A, *Klebsiella pneumoniae* growth; B, *Escherichia coli* growth; C, *Staphylococcus aureus* growth; D, *Pseudomonas aeruginosa* growth; E, *Staphylococcus* spp.; and F, no growth. S2 shows isolate sensitivity tests by use of the disc diffusion technique: A, *E. coli* sensitivity test; B, *S. aureus* sensitivity test; C, *K. pneumoniae* sensitivity test.

## Introduction

The prevalence of multidrug-resistant and virulent pathogens in healthcare settings poses a significant challenge to public health. Among these are the six pathogens represented in the acronym ESKAPE (* Enterococcus faecium *, *Staphylococcus aureus *, *Klebsiella pneumoniae *, *Acinetobacter baumannii *, *Pseudomonas aeruginosa *, and *Enterobacter* species), which exhibit multidrug-resistance and virulence, and a majority of nosocomial infections are attributed to these taxa [1, 2]. Since wounds offer a moist, warm, nutritive environment advantageous to microbial colonization, spread and infection, the ESKAPE pathogens usually have a high chance of leading to infection [3–6]. Skin is the first line of defence and is inhabited by various microbial assemblages which live on it [7, 8]. Bacterial infections, whether through contact with contaminated water, trauma, accidents, surgical operations or burns can result due to any breach in the skin surface [7]. A diverse array of microorganisms ranging from bacteria to fungi and parasites and viruses can infect wounds [9, 10]. These organisms can lead to the formation of wound biofilms, which further complicates wound healing as biofilms have increased antibiotic resistance [10].

Unfortunately, control of wound infections has become more challenging due to widespread bacterial antibiotic resistance and to a greater occurrence of infections caused by methicillin-resistant *S. aureus* (MRSA)*,* polymicrobial flora [11]. Although the development of antimicrobials in the 20th and 21st centuries transformed the treatment of human infections, the rapidly increasing antimicrobial resistance poses a threat to mitigating infections. Managing the increasing range of infections caused by microorganisms remains a dynamic risk due to antimicrobial resistance [12]. Hence the management of patient demands and increasing costs is leading to persistent infections and increasing deaths due to since the reduced effectiveness of antibacterial drugs [12]. Antimicrobial resistance is increasing in incidence due to the burden of using and misapplying antibiotics [13]. There has been an emerging desire for the development of new antibiotics that are more effective in the management of resistant bacteria [13], due to common bacteria developing resistance to recently discovered antimicrobials [13, 14]. The absence of new antibiotics to replace those that are no longer effective is given more urgency with the desire to guard the effectiveness of current medications, and advance and enact suitable methods to curb the rise and spread of antimicrobial resistance [15].

In developing countries, antimicrobial resistance poses a great threat to community health care, leading researchers to determine the resistance profile of antibiotics against bacteria. Nevertheless, the conclusions continue to produce different opinions on the effectiveness of some drug. For instance, studies in Libya [16, 17], as in Sudan [18], revealed sensitivity of isolates obtained from wounds at rates of 54 % with amikacin and 59 % with ciprofloxacin but resistance at rates of 81 % with vancomycin, 75 % with amoxicillin, 92 % with streptomycin, 45 % with tetracycline, 26 % with methicillin, 65 % with amoxicillin and 46 % with erythromycin. On the other hand, bacterial isolates obtained from wounds were highly resistant (96 %) to ciprofloxacin in Nigeria [19], which contradicts other findings.

The growing complication of antimicrobial resistance remains a disturbing problem worldwide, motivating boundless research and novel approaches to research on bacterial susceptibility and resistance to antibiotics is a priority subject, sometimes producing contradictory results. For instance, in wound management, Pokhrel *et al*. [19], and Gupta *et al*., [20] recognized that *S. aureus* isolates were therapeutically responsive to amoxicillin clavulanate, meropenem, clindamycin, ceftriaxone, piperacillin-tazobactam, ciprofloxacin, vancomycin, levofloxacin, linezolid, teicoplanin, imipenem, meropenem, amikacin and Levofloxacin but resistant to ampicillin, amoxicillin clavulanate, cotrimoxazole, doxycycline and cephalosporins. Other studies revealed high resistance to cephalosporins, amoxicillin clavulanate and imipenem [20, 21]. Hence, the present study evaluated bacterial isolates from patients with wound infections and drug susceptibility patterns were examined, with the goal of deciphering antibacterial resistance, from which some resistant isolates were identified based on commonly used antibiotics.

## Methods

We examined all samples received at the Medical Microbiology Laboratory Department of Jaramogi Oginga Odinga Teaching and Referral Hospital (JOOTRH), Kisumu county, Kenya, for the period of May to August 2022. Samples (wound pus/swab) were obtained from the patients who were seeking medicare at JOOTRH both in outpatient and in inpatient departments; patients referred to the hospital were not included in this study.

Following aseptic techniques, microscopy was performed to analyse the type and morphology of bacteria. The samples were then inoculated on blood agar (BA), MacConkey agar (MA) and chocolate agar (CA) following previously used and established standard protocols [22]. BA and MA plates were incubated aerobically, while CA plates were incubated in anaerobic conditions at 37 °C for 24 h. All isolates were identified by colony morphology, gram staining reaction and biochemical properties [22]. The antimicrobial susceptibility test of isolates was performed by the Kirby–Bauer disc diffusion method using the Clinical and Laboratory Standards Institute (CLSI) guidelines on Müeller–Hinton agar (MHA) plates (Mast Diagnostics) where 16 antimicrobials, i.e. penicillin (25 µg), ampicilin (10 µg), vancomycin (15 µg), cefoxitin (10 µg), cefuroxime (30 µg), oxacillin (15 µg), ciprofloxacin (10 µg), clindamycin (25 µg), co-trimoxazole (30 µg), tetracycline (15 µg), erythromycin (10 µg), gentamicin (30 µg), ceftriaxone (10 µg), cefepime (30 µg), imiperine (10 µg) and amikacine (30 µg) wer used, as outlined by the CLSI (2022) [23]. The isolated *S. aureus* were screened for methicillin resistance using cefoxitin discs (30 µg) as per standard guidelines provided by the CLSI [23]. MICs of ciprofloxacin and gentamicin were determined by the agar dilution method [24, 25] and the guidelines of the CLSI [23]. The tests were performed by making a series of antibiotic concentrations on MHA plates. *P. aeruginosa* ATCC 12934 and *S. aureus* ATCC 29213 were used as reference strains (controls). All the data were entered in SPSS version 20. Statistical analyses were done using the same software.

### Monitoring the presence of relevant virulence factors of *P. aeruginosa* spp*.*

#### Detection of haemolysin

Haemolysin production by the *P. aeruginosa* spp. isolates was detected following the protocols given by Benson *et al*. [26]. Beta-haemolytic activity was tested for on base agar (Himedia) supplemented with 7 % sheep erythrocytes for 18–24 h. Pure isolates were cultured on TSA, before streaking on BA and further incubated for 24 h at 37 °C. Zones of haemolysis around the colonies after 24 h indicated the ability of these bacteria to haemolyse red blood cells (RBCs) [27].

#### Detection of protease

To detect protease production by the *P. aeruginosa* spp., skim milk agar was used and a previously described protocol [26]. Briefly, two solutions (A and B) were made and used in this study. Solution A was prepared by adding 10 g skimmed milk to 90 ml distilled water, the volume was then completed to 100 ml, gently heated at 50 °C, then autoclaved and cooled to 50–55 °C.

Solution B was also prepared by adding 2 g of agar powder to 100 ml distilled water, mixed thoroughly, then autoclaved and cooled to 50–55 °C. Aseptically, 100 ml of solution A was mixed with 100 ml of solution B. The mixture was then poured into sterile Petri dishes, and then stored at 4 °C until use. This medium was used to detect the ability of the bacteria to produce protease [28]. The appearance of a cleared hydrolysis zone indicated a positive test [29].

#### Detection of lipase

Lipase production ability by *P. aeruginosa* isolates were determined by methods outlined by Elliot *et al*. [29]. Briefly, a single colony of an overnight growth was cultured on Rhan medium, and then incubated for 1–5 days at 37 °C. The appearance of a turbid zone around colonies on the fourth day indicated a positive result [26].

#### Detection of lecithinase (phospholipase)

To detect lecithinase, we followed a standard procedure [30]. One pure colony was cultured on the medium used for phospholipase activity assay followed by incubation for 1–3 days at 37 °C using established procedures [31]. The appearance of a white to brown elongated precipitated zone around colonies on the second day was considered a positive result [32].

### Validity and reliability

All experiments were conducted in triplicates that were independent of each other to validate reproducibility.

### Data analysis

Statistical analysis was performed using SPSS version 20. Data on socio-demographics were summarized by frequencies and percentages. All values of diameter zones of inhibition are reported as mean±se.

## Results

Of 135 samples processed, 73 (54.1 %) showed bacterial growth (supplementary data S1. A–F) and 62 (45.9 %) did not show growth. Among growth positive cases, 37 (27.4 %) were Gram-positive while 36(26.7 %) were Gram-negative. Higher rates of growth were found in male patients, with 23 (29.1 %) Gram-positive cocci and 26 (32.9 %) Gram-negative cocci, than in female patients, with 14 (25.5 %) Gram-positive isolates and 10 (18.2 %) Gram-negative isolates; this was found to be statistically significant (*P*<0.075). Among total growth, the highest growth rate was found in age groups >45 years in both Gram-positive and Gram-negative bacteria, 14 (26.9 %) and 12 (23.1 %) respectively. Least growth was found in the age groups <5 years and 25–34 years (Table 1).

**Table 1.** Sociodemographic characteristics of patients involved in the study for the period of May to August 2022

Characteristic	Total (*N*, %)	Gram-positive (*n*, %)	Gram-negative (*n*, %)	No growth	*P* value
**Age category**					
< 5 years	12 (9 %)	1 (8.3 %)	6 (50 %)	5 (41.7 %)	0.199
6–14 years	24 (17.9 %)	9 (37.5 %)	6 (25 %)	9 (37.5 %)	
15–24 years	15 (11.2 %)	8 (53.3 %)	2 (13.3 %)	5 (33.3 %)	
25–34 years	16 (11.9 %)	2 (12.5 %)	6 (37.5 %)	8 (50 %)	
35–44 years	15 (11.2 %)	3 (20 %)	4 (26.7 %)	8 (53.3 %)	
>45 years	52 (38.8 %)	14 (26.9 %)	12 (23.1 %)	26 (50 %)	
**Gender**					
Female	55 (41 %)	14 (25.5 %)	10 (18.2 %)	31 (56.4 %)	0.075
Male	79 (59 %)	23 (29.1 %)	26 (32.9 %)	30 (38 %)	
**Total**	**134**	**37** (27.6 %)	**36** (26.9 %)	**61** (45.5 %)	

Out of the total wound swab samples analysed, 71 (52.6 %) were from swab samples while 64 (47.4 %) were aspirate samples and a higher percentage growth was observed on aspirate than swab samples (Table 2). Of the 73 (54.1 %) growth-positive wound swab samples, 37 (27.4 %) provided Gram-positive bacteria, while 36 (26.7 %) provided Gram-negative bacteria. Among the Gram-positive isolates after biochemical testing, *S. aureus* [28 (20.7 %)] was the most predominant followed by *Staphylococcus* spp. [9 (6.7 %)] while among the Gram-negative isolates on biochemical testing the most predominant organism was *Klebsiella* spp. [20 (14.8 %)] followed by *P. aeruginosa* [10 (14.8 %)] and *E. coli* [6 (4.4 %)], as shown in Table 2.

**Table 2.** Distribution of bacterial strains among sample types

Sample	Total (*N*, %)	* S. aureus *	* Staphylococcus* spp.	* Klebsiella* spp.	* P. aeruginosa *	* E. coli *	No growth
Swab	71 (52.6 %)	9 (12.7 %)	3 (4.2 %)	3 (4.2 %)	1 (1.4 %)	0 %	55 (77.5 %)
Aspirate	64 (47.4 %)	19 (29.7 %)	6 (9.4 %)	17 (26.6 %)	9 (14.1 %)	6 (9.4 %)	7 (10.9 %)
**Total**	**135** (100 %)	**28** (20.7 %)	**9** (6.7 %)	**20** (14.8 %)	**10** (7.4 %)	**6** (4.4 %)	**62** (45.9 %)

Among 37 (27.4 %) Gram-positive isolates, 28 (20.7 %) *S *. *aureus* resistance patterns revealed that the most effective antibiotics were cotrimoxazole [13 (48.1 %)] followed by clindamycin [7 (25.9 %)] and erythromycin [7 (25.9 %)] while lower resistance to cotrimoxazole [3 (37.5] %) was observed with *Staphylococcus* spp. Among 36 (26.7 %) Gram-negative isolates, 20 (14.8 %) *Klebsiella* spp. resistance patterns revealed that the most effective antibiotic was tetracycline [11 (61.1 %)] followed by gentamicin at [9 (50 %)] while for *P. aeruginosa* four isolates showed multi-drug resistance (MDR) to commonly used antibiotics, while for *E. coli* the isolates were sensitive to almost all of the common antibiotic used in the facility for their management, as shown in Table 3 and Supplementary data 2. A–C.

**Table 3.** Antibiotic resistance patterns of isolated pathogens

Gram-positive	Cefoxitin	Oxacillin	Vancomycin	Penicillin	Cotrimoxazole	Clindamycin	Tetracycline	Gentamicin	Erythromycin
* S. aureus *	3 (11.1 %)	2 (7.4 %)	2 (7.4 %)	5 (18.5 %)	13 (48.1 %)	7 (25.9 %)	5 (18.5 %)	4 (14.8 %)	7 (25.9 %)
* Staphylococcus* spp.	2 (25 %)	0 %	1 (12.5 %)	1 (12.5 %)	3 (37.5 %)	1 (12.5 %)	1 (12.5 %)	1 (12.5 %)	1 (12.5 %)
**Gram-negative**	**Cotrimoxazole**	**Ciprofloxacin**	**Cefuroxime**	**Ceftriaxone**	**Cefepime**	**Imipenem**	**Ampicillin**	**Gentamicin**	**Amikacin**
* Klebsiella* spp.	5 (27.8 %)	4 (22.2 %)	1 (5.6 %)	3 (16.7 %)	3 (16.7 %)	6 (33.3 %)	11 (61.1 %)	9 (50 %)	2 (11.1 %)
* P. aeruginosa *	0 %	1 (10 %)	2 (20 %)	1 (10 %)	1 (10 %)	3 (33.3 %)	2 (20 %)	2 (20 %)	2 (20 %)
* E. coli *	0 %	0 %	0 %	0 %	0 %	1(16.7 %)	0 %	0 %	0 %

Of 10 *P. aeruginosa* isolates, four strains were MDR, with one strain was resistant to three classes of antibiotics (12583), two strains were resistant to five classes of antibiotics (13642 and 14421) and one strain was resistant to six classes of antibiotics (11985).

This study investigated the production of various virulence enzymes such as protease, phospholipase, lipase and haemolysin on the four *P. aeruginosa* isolates which showed MRD and were found to be resistant to common antibiotics used for its management within the study area. Of the 10 isolates four showed resistance to different antimicrobial classes, and one of which, isolate 14421, was able to produce all types of the virulence enzymes. It was also confirmed that isolate 12583 was able to produce all enzymes except phospholipase while isolate 11985 was also capable of producing all except lipase. Lastly, isolate 13642 was not able to produce any of the virulence enzymes, as shown in Table 4.

**Table 4.** Detection of some virulence factors of *P. aeruginosa
*

Isolate	Haemolysin	Protease	Lipase	Phospholipase
12583	+	+	+	−
13642	−	−	−	−
14421	+	+	+	+
11985	+	+	−	+

Key: +, positive; –, negative.

## Discussion

Antibiotic resistance is a global health concern, necessitating ongoing monitoring. This study is a response to the pressing need for continuous surveillance of antibiotic resistance trends, especially in the context of chronic wound infections, which remain a public health concern. The study focused on patients with chronic wounds seeking medical care at Jaramogi Oginga Odinga Teaching and Referral Hospital (JOOTRH) in Kenya. By collecting and analyzing wound samples, the research aimed to uncover the prevalence and patterns of antibiotic resistance among bacterial pathogens in this specific population. The findings were both particularly interesting and alarming. Out of the 135 samples collected from patients with wound infections, 73 (54.1%) exhibited positive bacterial growth, indicating a substantial burden of microbial infections. The isolates included a range of bacteria, with *Staphylococcus aureus *, *Klebsiella spp.*, *Pseudomonas aeruginosa* and *Escherichia coli* being the most prevalent. These pathogens are known to be responsible for various wound infections and can pose significant challenges in treatment. In similar studies conducted previously [22] on antibiotic susceptibility patterns of bacterial isolates causing wound infection among patients visiting two hospitals in India [24], positive growth rates were 44.9–50%. Separate studies [25, 33] in a tertiary hospital in South Africa showed more than 90 % growth emphasizing the eminent concern. The positivity rate was higher in male patients for both Gram-positive and Gram-negative isolates, 23 (29.1 %) and 26 (32.9 %) respectively. A similar study [34] agreed with our findings. The relatively higher percentage of male patients might be due to active involvement of males in outdoor activities, including agricultural work, resulting in high possibility of infection and prevalence of high rates of accidents [26]. Our findings underscore the concern of a significantly elevated positivity growth rate, potentially affecting the wound healing process, particularly since many samples contained multiple isolates. Conversely, despite the precautions taken in such studies, it remains uncertain whether some cases of culture-negative results can be ascribed to non-infectious bacteria, errors in sample collection, transportation challenges, delays in processing, or the prior use of antibiotics by patients.

Further, the data herein revealed intriguing insights into the prevalence of wound infections and antibiotic resistance patterns in different age groups. We found the highest rate of positivity in the age group >45 years, contrary to previous studies that reported the highest growth rate in the 21–30 years age group [26, 33]. This disparity might be attributed to the elevated presence of older individuals in our study, potentially resulting from increased activity and accidents leading to hospital admissions in this age category. From the total bacterial isolates, 37 (27.6 %) were Gram-positive and 36 (26.9 %) were Gram-negative bacteria. This result did not match with previous studies [21–26, 29–35] which showed Gram-negative bacteria being predominant since their peptidoglycan layer is much thinner than that of Gram-positive bacilli, and they are harder to eradicate because when their cell wall is disturbed they release endotoxins that can worsen patient symptoms [24]. Our observation of Gram-negative bacteria beinmg the most common in wound infections differs from other studies in Nigeria reporting Gram-positive bacteria to be predominant [29]. Among the Gram-negative pathogens, *Klebsiella* spp. was the most predominant [20(14.8 %)]; the pathogenicity of *K. pneumoniae* is mediated by several virulence factors that allow it to evade host innate immune responses. These factors include the capsule, lipopolysaccharide, adhesions, iron acquisition systems, resistance to serum and biofilm formation [34]. Among Gram-positive taxa, *S. aureus* was the predominant isolate [28 (20.7 %)] and the most predominant among the total. *S. aureus* pathogenicity is mediated by bacterial components and secreted virulence factors such as surface-associated adhesions, capsular polysaccharide (CP) and exotoxins [29]. This finding did not match a similar study conducted [32], where *P. aeruginosa* was the most predominant bacterium (29.9 %) among the total isolates, which may be due to the permeability barrier afforded by its Gram-negative outer membrane, while *S. aureus* was predominant (27.5 %) among Gram-positive isolates. In a previous study [32], Gram-negative bacilli constituted 66 % of all the pathogens with *P. aeruginosa* as the most frequent (19 %). A higher prevalence of *S. aureus* among Gram-positive bacteria and the most predominant isolate among the total seen in this study has also been reported by other researchers [10, 36],. These results shed light on the diversity of wound infections and the potential regional variations in bacterial prevalence, emphasizing the need for tailored treatment strategies. Moreover, they underscore the persistent challenge of antibiotic resistance and the importance of responsible antibiotic use to combat these infections effectively.

In the susceptibility patterns towards Gram-positive isolates, *S. aureus* isolates showed an alarming resistance to cotrimoxazole (48.1 %), a commonly used antimicrobial drug in wound management. Studies have shown that *S. aureus* can become drug-resistant either via genetic mutations on DNA gyrase or through reduced expression of outer membrane proteins reducing drug accumulation [53]. The findings align with earlier research, corroborating resistance rates of 47% and 49% to cotrimoxazole [37–39] . Cotrimoxazole has traditionally served as a first-line treatment for wound infections and has been extensively prescribed for gastrointestinal, respiratory, urinary, and skin infections. Its mechanism of action involves targeting a DNA gyrase subunit, crucial for bacterial DNA synthesis [39]. Furthermore, it is frequently administered to immunocompromised patients, such as those with HIV, which has a prevalence of 16.3% [Bibr R38]in the study area [38]. The widespread usage of cotrimoxazole, its affordability, and its accessibility without a prescription likely contributed to the emergence of resistance, as observed in our study. This underscores the urgent need for prudent antibiotic use to combat the growing challenge of antimicrobial resistance.

A notable occurrence of resistance was observed among *S. aureus* isolates, with a resistance rate of 25.9% against erythromycin. Erythromycin is a broad-spectrum, bacteriostatic antibiotic effective against various Gram-positive bacteria. Its mechanism of action involves irreversible binding to a receptor site on the 50S subunit of the bacterial ribosome, thereby inhibiting peptidyl transferase and preventing the transfer of amino acids to growing peptide chains, ultimately hindering protein synthesis. Until late 2018, erythromycin was the primary treatment for conditions like typhoid and Salmonella infections [32, 35, 40]. However, because of resistance and safety issues, it is no longer the first-line treatment in enteritis. In low-income countries, it is still widely used, as it is not expensive and is readily available [27]. Erythromycin has been recommended by the World Health Organization (WHO) for the treatment of wound infections in both children and pregnant women [41]. The high resistance observed may be explained by its frequent usage for the treatment of severe coughs and other infectious diseases. Also, erythromycin resistance was found to be due predominantly to the presence of an Erm (B) methylase. Other reports have indicated that *S. aureus* isolates are resistant to erythromycin [37]. A similar incidence was observed for other pathogens causing diarrhoea in a study conducted in the study area [42, 43].

Additionally, *Klebsiella spp.* demonstrated a notably high resistance rate of 61.1% to ampicillin, exceeding that of gentamicin at 50%. Ampicillin targets penicillin-binding proteins anchored in the bacterial cell membrane, essential for cell wall cross-linking. Klebsiella spp. isolates in previous wound infections have been recognized for their resistance to ampicillin, gentamicin, and imipenem [44]. There has been reported to be an emergence of *P. aeruginosa* isolates resistant to ampicillin (33.3 %), gentamicin, amikacin and ceftriaxone (all at 20 %), with 10 isolates resistant to this antimicrobial, which has been primarily associated with the presence of Inc C conjugative plasmids [41], hence leading to four isolates being found to be MDR. Among the MDR isolates, two showed resistance to three antimicrobial classes, one resistance to four antimicrobial classes, two resistance to five classes and finally one resistance to six antimicrobial classes. Studies on MRSA have shown their wide variation. Naik and Deshpande [45, 46] showed an 8.0 % prevalence of MRSA, which agrees with our study. All *E. coli* isolates were sensitive towards all the antibiotics used except lower resistance to imipenem, thus confirming the higher efficiency of these agents against *E. coli* isolates at Kisumu County.

Surprisingly, of four isolates of *P. aeuruginosa*, three (75 %) (12583, 14421 and 11985) were able to produce haemolysin, a finding that did n’t conform with a previous study [28] which showed 100 % isolates producing the enzyme. As stated previous, purified haemolysin can cause fluid accumulation [32]; in contrast to the watery fluid produced in response to Cholera Toxic (CT), the accumulated fluid produced in response to haemolysin is invariably bloody with mucus [32]. Also, of the four isolates, two (50 %) (12 583 and 14421) had the ability to produce lipase. Our findings also concur with other studies, which showed that 50 % f isolates obtained in the study had the ability to produce lipase [16]. Lipase enzymes catalyse the hydrolysis of the ester bonds of triacylglycerols and may have a critical role in *P. aeruginosa* pathogenicity or nutrition acquisition. The production of excess lipase allows bacteria to penetrate fatty tissue with the consequent formation of abscesses [45, 47]. The production of these enzymes by the isolates may reflect the presence of genetic organization of a discrete genetic element, which encodes three genes responsible for producing proteases, lipases and phospholipase. This organization could be a part of a pathogenic island, encoding a product capable of damaging host cells and being involved in nutrient acquisition [48]. These findings underscore the urgent need for responsible antibiotic use and the development of more effective antibiotics to address chronic wound infections and the growing antibiotic resistance threat.

## Conclusion and recommendations

AMR isn't merely an impending crisis; it stands as an immediate global emergency, imperiling our capacity to effectively combat infections, conduct surgical procedures, and manage diseases that were once easily treatable. It represents a silent pandemic, stemming from the excessive and inappropriate use of antibiotics, inadequate hygiene and sanitation practices, and a dearth of innovative antimicrobial solutions. The findings of this study underscore a deeply troubling trend in antibiotic resistance among both Gram-positive and Gram-negative bacteria, resulting in treatment failures. These resistant strains specifically generate various virulent enzymes, including haemolysin, lipase, protease, and phospholipase C, culminating to bacterial resistance. Furthermore, despite inhibitory measures, the study's results reveal the capacity of these antibiotic-resistant strains to create biofilms. As AMR escalates across a wide spectrum of microorganisms, there is an urgent, global imperative to enhance antimicrobial treatments and pioneer innovative strategies for developing new antimicrobials. This entails utilizing innovative tools such as gene-mining applications and high-throughput genetic sequencing, along with the integration of computational tools and artificial intelligence to identify novel antimicrobial agents. Moreover, the collection of clinical data on AMR assumes paramount importance for the monitoring of resistance trends and in the development of superior antimicrobial drugs. This coupled with the adoption of forward-thinking approaches like artificial intelligence will expedite the discovery of new drugs and prioritize the issue of mounting antibiotic resistance in bacteria, including within the realm of chronic wound management.

## Supplementary Data

Supplementary material 1
