# Supplementary material for: Monitoring the battleground: exploring antimicrobial resistance and virulence factors in wound bacterial isolates
Source: Access Microbiol. 2023 Nov 9;5(11):000613.v6. doi: 10.1099/acmi.0.000613.v6 (PMC10702375; doi:10.1099/acmi.0.000613.v6)

Supplementary Data

Supplementary data 1: Isolate growth on Blood Agar Media; **A-** *K. Pneumoniae* growth, **B-** *E. coli* growth**, C-** *S. aureus* growth, **D-** *P. aeruginosa* growth, **E-** *Staphylococcus spp.* and **F-** No growth.

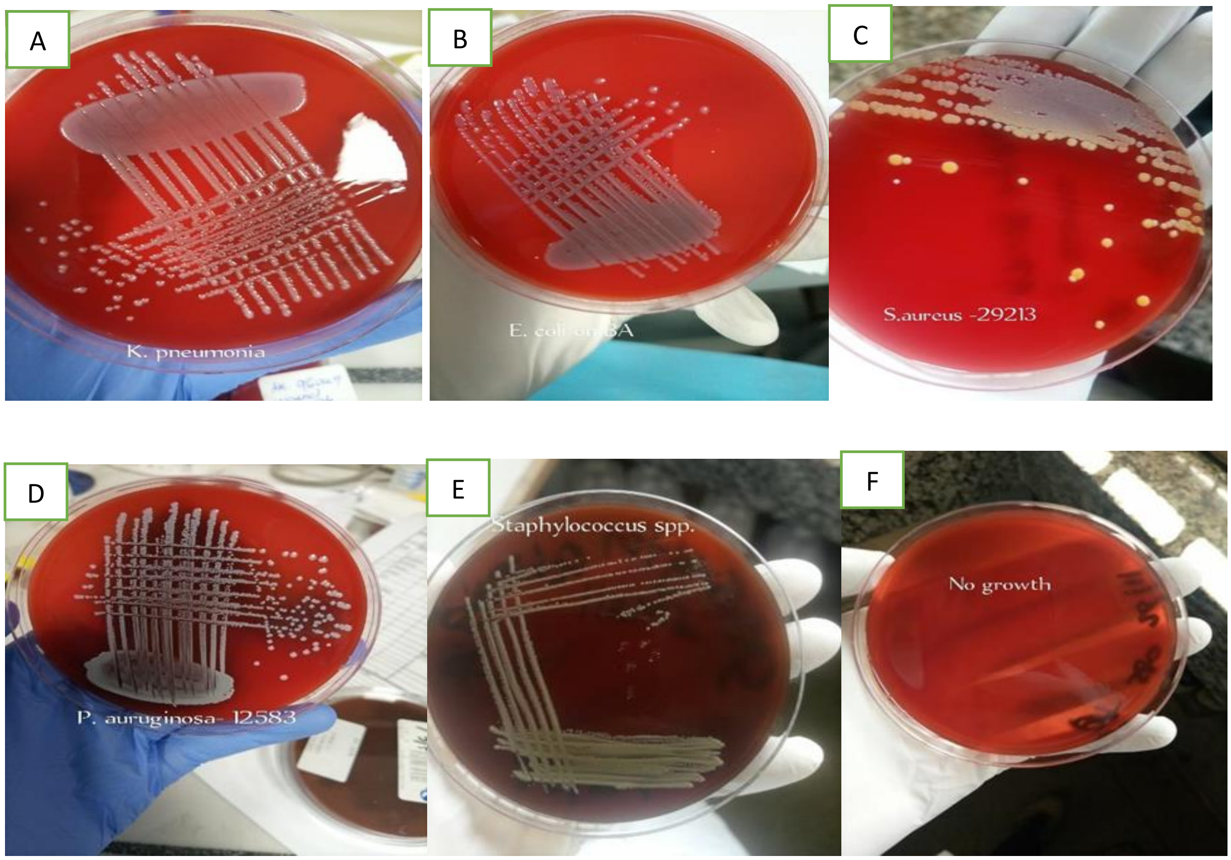

Supplementary data 2: Isolate sensitivity test by use of disk diffusion technique; **A-** *E. coli* sensitivity test, **B-** *S. aureus* sensitivity test, **C-** *K. pneumoniae* sensitivity test.

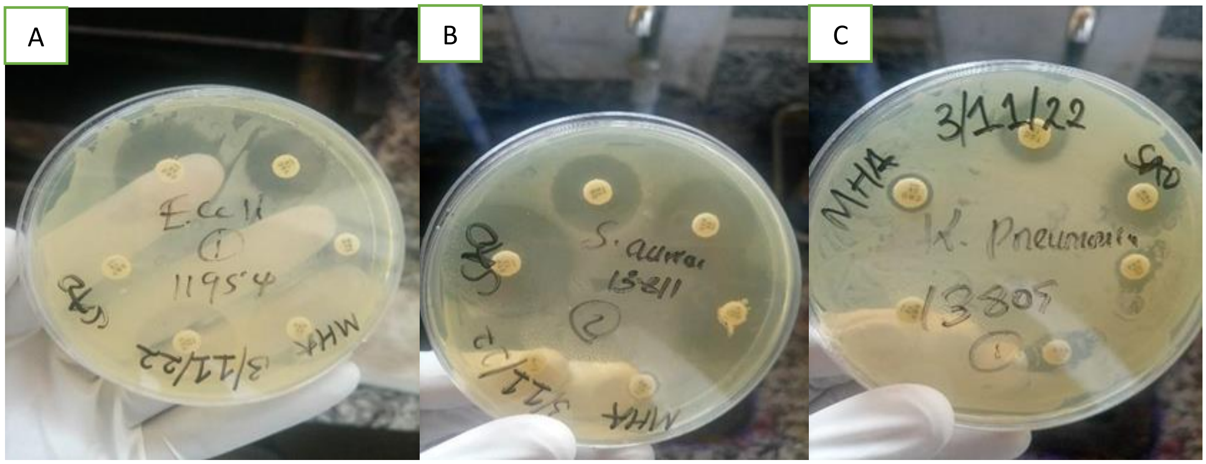

Supplement: Supplementary material 1 [file acmi-5-613.v6-s001.pdf]
